# A Systematic Review of Tablet-Based Interactive Distraction as a Preoperative Anxiolytic in Pediatric Patients Undergoing Same-Day Procedures

**DOI:** 10.7759/cureus.60274

**Published:** 2024-05-14

**Authors:** Chance J Aplanalp, Randall Hansen, Alex Otto, Suporn Sukpraprut-Braaten, Hallie Baker, Tanner Aldridge, Jaxon Davis, Blake Hansen

**Affiliations:** 1 Otolaryngology - Head and Neck Surgery, Kansas City University of Medicine and Biosciences, Joplin, USA; 2 Otolaryngology - Head and Neck Surgery, Freeman Health System, Joplin, USA; 3 General Medical Education, Freeman Health System, Joplin, USA; 4 Medicine, Kansas City University of Medicine and Biosciences, Joplin, USA; 5 Medicine, University of Washington, Spokane, USA

**Keywords:** anxiety reducing strategies, tablet-based distraction, adeno-tonsillectomy, tympanostomy tube, pre-operative anxiety, distraction techniques, tablet

## Abstract

Evidence shows tablet-based interactive distraction (TBID) is effective as a preoperative anxiolytic in pediatric patients. TBID involves age-appropriate video games that have been preloaded onto a tablet (TAB) and subsequently given to a pediatric patient before the administration of anesthesia. The purpose of this study is to provide a comprehensive analysis of previous studies that have investigated the use of TBID to minimize preoperative anxiety.

The literature criteria for this systematic review included randomized controlled trials and prospective studies that used TBID as a method to reduce preoperative anxiety in pediatric patients aged 1-12 years. Data extraction concentrated on the patient population to which the TABs were introduced, the method of TAB administration, how anxiety was evaluated, who completed the evaluations, and the results of each publication. This chosen data set is to systematically understand if TBID is effective and to identify the most practical ways to implement TBID. Collected data from the selected publications were entered into a table.

For this systematic review, 27 publications from 2006 to 2023 were screened for eligibility. These studies were selected using a combination of MeSH terms and a Title-Abstract filter in PubMed, Embase, and Scopus. These data represented 475 total patients (T) and 249 patients who implemented TAB use. The other 226 patients were used as various control groups. The outcome of each study is summarized and placed into a table. This study is expected to provide an overall assessment of the effectiveness of TBID and proposed guidelines for clinicians to incorporate TAB use into preoperative protocols. The time to give the TAB to the children impacts its efficiency.

This review accentuates the effectiveness of utilizing TBID to mitigate preoperative anxiety in pediatric patients based on a comprehensive analysis of multiple prior studies conducted in diverse healthcare settings, including pediatric hospitals and surgical centers. TAB use demonstrated an effective reduction in perioperative anxiety, emergence of delirium, and time to discharge, increasing parental satisfaction compared to midazolam. These results are likely replicable across a broader range of clinical settings, provided the intervention parameters, such as the timing of TAB introduction and the personalization of content to patient interests, are carefully adapted to each situation. The anxiety evaluations of patients using TBID varied based on the evaluator. Therefore, future research should analyze if perceived anxiety in patients using TABs is consistent or not among the evaluators. The impact of this TBID review has the potential to set a new benchmark for managing pediatric preoperative anxiety, with significant implications for healthcare quality and patient satisfaction.

## Introduction and background

For pediatric patients, the preoperative rooms can be cold, intimidating, and filled with individuals wearing matching scrubs and masks. Despite her reassurances from parents or loved ones, the unknown of the upcoming procedure weighs heavily on these patients. Preoperative pediatric anxiety has been linked to adverse outcomes, unsafe inductions, sleep disturbances, and lower patient satisfaction. Traditionally, benzodiazepines, beta-blockers, and opioids have been used preoperatively to minimize preoperative anxiety. Although these medications reduce preoperative anxiety, several studies suggest that the emerging approach of tablet-based interactive distraction (TBID) offers a novel and promising alternative equally effective as a preoperative anxiolytic in pediatric patients [1-7]. TBID involves age-appropriate video games that have been preloaded onto a tablet (TAB) and given to a pediatric patient before administering anesthesia. The purpose of this study is to provide a comprehensive analysis of studies that have investigated the use of TBID as a means of minimizing preoperative anxiety. This systematic review is expected to provide a synopsis of the effectiveness of TBID and proposed guidelines that clinicians can utilize as they incorporate TAB use into preoperative protocols.

One of the significant challenges faced when implementing TBID is the degree of uncertainty around when TAB use is indicated or in what context TAB use could benefit the patient. Additionally, the published literature regarding TBID has yet to reach a consensus on patient age range or specific timing when the TAB is introduced. Inconsistencies that exist within TBID literature are how anxiety is being assessed and who is evaluating the anxiety of the patient. Specific questionnaires that evaluate anxiety levels have been accepted and will be mentioned later; nevertheless, pediatric populations pose another level of difficulty when assessing anxiety levels [8]. Therefore, a lack of guidelines exists about the use of TBID, and as such, there is a paucity of information about its use, including but not limited to indications, patient population, TAB timing before the procedure, and the interpretation of the benefits of TAB use.

## Review

Methodology

The literature criteria for this systematic review included randomized control trials and clinical trials that used TBID to reduce preoperative anxiety. PubMed, Embase, and Scopus were used as the primary databases. The MeSH terms were first used to initiate the search. Subsequently, the Title-Abstract filter was utilized to narrow down the results to the review's targeted population. To conclude the database search, additional filters were applied that included randomized control trials and clinical trials that were carried out in English and that focused on infants (1-23 months), preschool children (2-5 years), and children (6-12 years) only. The search query is shared below:

"(((((((((("Pediatrics"[Mesh]) OR "Child"[Mesh]) OR "Child, Preschool"[Mesh]) OR "Infant"[Mesh]) AND (tablet[Title/Abstract])) OR (computer[Title/Abstract])) OR (hand-held[Title/Abstract])) AND (distraction[Title/Abstract]) ) NOT (virtual reality[Title/Abstract])) NOT (cartoons[Title/Abstract])) NOT (videos[Title/Abstract]) Filters: Clinical Trial, Randomized Controlled Trial, English, Infant: 1-23 months, Preschool Child: 2-5 years, Child: 6-12 years."

**Figure 1:**
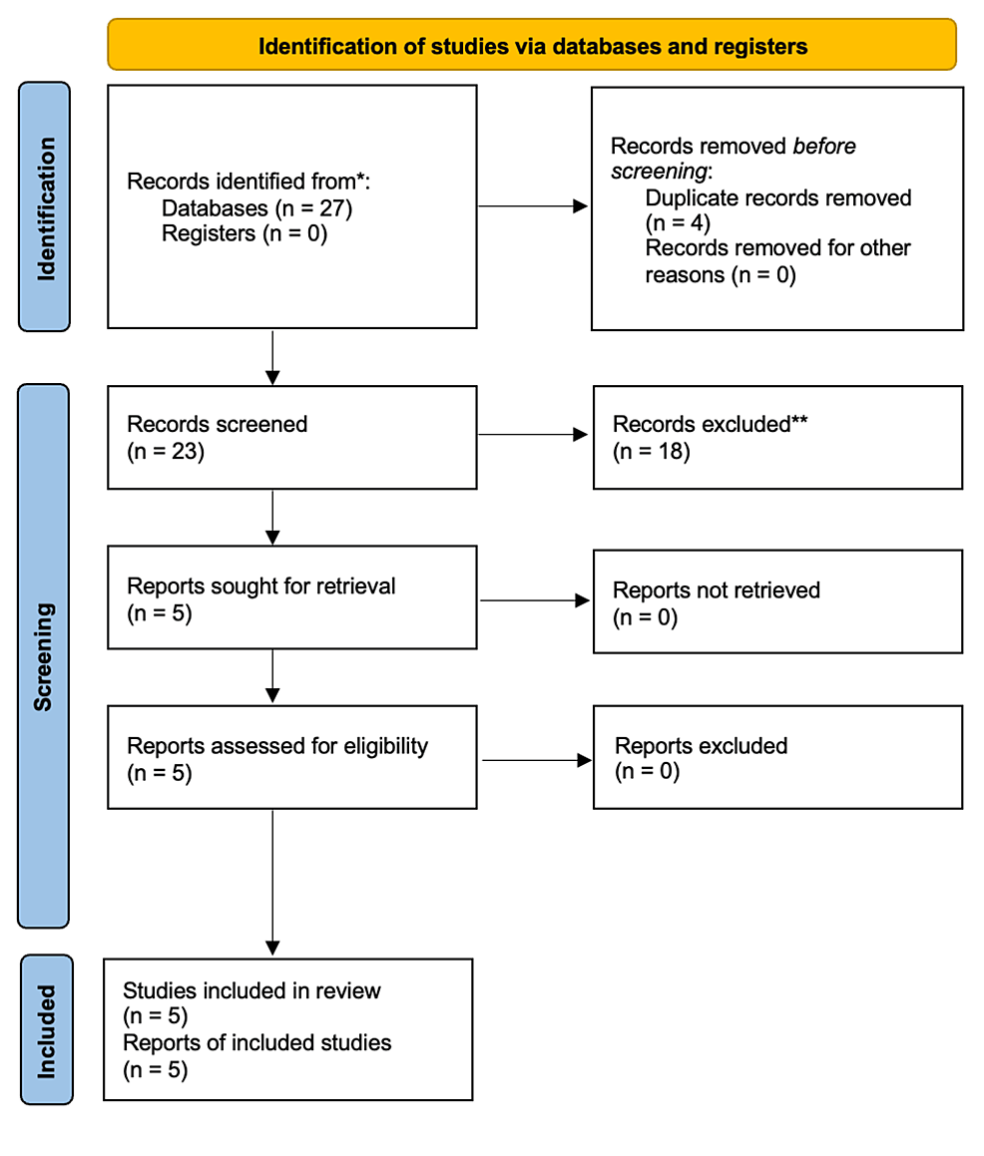
PRISMA for systematic review. PRISMA, Preferred Reporting Items for Systematic Reviews and Meta-Analyses

The selected publications needed to focus on pediatric patients aged 1 to 12 years undergoing same-day surgical procedures. If the literature only involved a selective age range, such as *only 2-5-year-olds*, the paper was excluded. Whether TBID was being compared to another form of treatment or if anxiety levels after TAB administration were measured alone, the collected data must focus on the impact of TAB use on anxiety levels. A standardized Preferred Reporting Items for Systematic Reviews and Meta-Analyses (PRISMA) diagram is used in Figure 1, illustrating the sequence of events that determined which studies were used in this review [9].

Data extraction concentrated on the patient population to which the TABs were introduced, the method of TAB administration, how anxiety was evaluated, who completed the evaluations, and the results of each publication. This chosen data set was used to systematically understand if TBID is effective and to identify the most practical implementation of TBID. Collected data from the selected publications was entered into a table. Each column is organized by the following: author and year of publication, study age group, device used and how games were selected, how and when the TABs were introduced, who performed the evaluation, focus of the evaluator, methods of anxiety assessment, and the overall results. 

In addition, secondary outcomes from each study are also mentioned in the table. These supplementary results are broad but demonstrate overlapping features found within each study. 

Quality assessment and risk of bias

The cale for the Quality Assessment of Narrative Review Articles (SANRA) was used to assess the risk of bias in the papers included in this review [10]. This assessment generates a score from 0 to 12, with 12 indicating the highest quality of research. The average score among the five studies used was calculated to be 9.0. This score suggests that the quality of the literature involved in this review is high. Stewart et al., Marechal et al., and Patel et al. mentioned that the only possible bias in their study was observer bias. This is possible because the nurses who completed the Modified Yale Preoperative Anxiety Scale (m-YPAS) scoring were aware of which group the participant belonged to. Also, none of the studies used in this review mentioned conflicts of interest or notable financial disclosures.

Outcomes measures and effect direction plot 

Due to the significant heterogeneity of the survey used, control groups, and outcomes from the articles used in this review, a meta-analysis was implausible. Therefore, a narrative synthesis was conducted in accordance with Cochrane's Synthesis Without Meta-analysis (SWiM) guidelines [11]. Instead, data were evaluated using a standardized narrative analysis. An effect direction plot was utilized to illustrate the result of the narrative data synthesis with the associated legend to guide plot significance [12] (Table 1). 

**Table 1:** The effect direction plot Effect direction: upward arrow (▲) = positive health impact; downward arrow (▼) = negative health impact; sideways arrow (◄►) =  no change/mixed effects/conflicting findings. Sample size: Final sample size (individuals) in the intervention group: Large arrow ▲ >300; medium arrow ▲ 50-300; small arrow ▲ <50. Study quality (denoted by row color): green, low risk of bias; amber, some concerns; red, high risk of bias. Study design: RCT and CRCT RCT, randomized controlled trial; CRCT, cluster randomized controlled trial

Study	Study design	Preoperative anxiety	Emergence of delirium	Overall/Caregiver satisfaction	Compared to midazolam	Overall anxiety
Gezginci et al, 2021 [13]	RCT	◄►	▲	▲	◄►	▲
Marechal et al., 2017 [14]	RCT	◄►	▲	▲	◄►	▲
Stewart et al., 2019 [15]	RCT	▲	▲	◄►	▲	▲
Seiden et al., 2014 [1]	RCT	▲	▲	▲	▲	▲
Patel et al., 2016 [2]	RCT	▲	◄►	◄►	▲	▲

Results

Twenty-seven publications from 2006 to 2023 were screened and assessed for eligibility for this systematic review. These studies were selected using a combination of MeSH terms, a Title-Abstract filter, and other filters mentioned in the methods section of this review. Four of the original identified articles were excluded due to duplications. Screening and assessment for study eligibility and reasons to exclude studies were based on the previously mentioned criteria. The selected literature must have implemented TBID as a preoperative anxiolytic in pediatric patients ages 1 to 12. For this reason, 18 of the remaining 23 studies were excluded from this review. Of these five studies, four were randomized control trials, and one was a prospective cohort study using TBID. Data was gathered from 475 total patients (T), 249 of whom had implemented TAB use. The remaining 226 patients in this review did not receive a TAB during their evaluation but were part of control or comparison groups. The comparison and control groups ranged from no intervention to oral midazolam (MDZ) and parental presence.

Table 2 summarizes the study characteristics extracted from each publication for this systematic review. This includes the total sample size (T), TAB use sample size, the age range of patients, how the TAB was used and presented, the time frame before the procedure the TAB was given, what was evaluated, who performed the evaluation, and the results. Although the main focus was to systematically review the effects of TBID on preoperative anxiety levels, some of the studies go beyond this scope and discuss secondary results mentioned in Table 2. These include patient and parental satisfaction, delirium emergence, post-anesthesia stay length, and time to discharge.

**Table 2:** Summary of the studies involved in the systematic review. T, total patients; TAB, patients in the tablet group

Article(Author and year)	Study population (age group)	Tool	Length of the study and methods	Who evaluated the patient, and what was assessed?	Results
(1) Gezginci et al. (2021) [13]	Children (70T-35 TAB) aged from 8 to 12 years who were undergoing circumcision	Tablet: Children could choose from three games suitable for their age.	Children selected their game 5-10 minutes before surgery. The children started playing their game five minutes before the procedure.	The blinded evaluator used a Numeric Rating Scale (pain) and the State-Trait Anxiety Scale (anxiety); satisfaction was also assessed.	Pain levels and anxiety after the procedure were significantly reduced in the TAB group, and overall satisfaction scores were also higher. Preoperative anxiety was not significantly reduced in the TAB group.
(2) Stewart et al. (2019) [15]	Children (102T - 51 TAB) aged from 4 to 12 undergoing outpatient surgery	iPad mini with age-appropriate games were selected by the child life specialist.	The tablet was given one minute before separation from caregivers. Tablet use was compared to midazolam use (0.3 mg/kg orally).	Pre-op and PACU nurses, a child life specialist, and the director of anesthesia assessed the patients using the Modified Yale Preoperative Anxiety Scale-Short Form (m-YPAS-SF) and the Pediatric Anesthesia Emergence Delirium (PAED) Scale.	The iPad was significantly more effective than oral midazolam in reducing preoperative anxiety, delirium emergence, and the length of post-anesthesia stay. No statistical difference was found in caregiver satisfaction.
(3) Marechal et al. (2017) [14]	Children (118T - 60 TAB) aged from 4 to 10 presenting for outpatient surgical procedures	Tablet: Children selected games based on their age and preferences	A tablet was provided 20 minutes before anesthesia. Tablet use was compared to midazolam use (0.3 mg.kg orally).	Patient anxiety was evaluated by independent psychologists using the modified Yale Preoperative Anxiety Score (m-YPAS), while parent anxiety and the State-Trait Anxiety Inventory (STAI).	Tablet use increased nurse and parental satisfaction. There was no significant reduction in anxiety at mask induction nor parental separation with the tablet use compared to midazolam use, but mean m-YPAS in the tablet group over four measurements was reduced.
(4) Seiden et al. (2014) [1]	Children (108T - 57 TAB) aged from 1 one 11 years undergoing general anesthesia for minor outpatient surgery	Tablet: Specific game choices were provided based on age appropriateness.	The tablet was given to children one minute before parental separation and used until induction. Specific game choices were provided based on age appropriateness. Tablet use was compared to midazolam use (0.5 mg/kg orally).	Clinicians assessed anxiety using m-YPAS. They also evaluated emergence delirium (PAED), parental satisfaction, and time to post-anesthesia care unit (PACU) discharge.	Tablet use demonstrated an effective reduction in perioperative anxiety, emergence of delirium, and time-to-discharge, increasing parental satisfaction compared to midazolam.
(5) Patel et al. (2006) [2]	Children (112T - 38 TAB) aged from 4 to 12 years undergoing outpatient surgery	Handheld video games: The child selected a video game from 10 options.	The tablet was used before the surgery; children were allowed to play through the introduction of the anesthesia mask. Tablet use and parent presence were compared to a midazolam group (0.5 mg/kg orally).	An independent observer evaluated anxiety at two distinct times during the surgical course using m-YPAS.	Handheld video game use did not show an increase of anxiety at induction of anesthesia compared to midazolam and parent presence (PP), which did.

An inconsistency across all of the featured studies is the evaluator assessing the patients. Moreover, Table 2 also displays that some of these studies included multiple individuals filling out the evaluations. These evaluators ranged from preoperative nurses, PACU nurses, child life specialists, the medical director of anesthesia, blind evaluators, clinicians, independent observers, and independent psychologists. Four research groups used a standardized assessment for evaluating anxiety: versions of the Yale Preoperative Anxiety Score (YPAS) and the State-Trait Anxiety Inventory (STAI). Gezginci et al. elected to use a numeric scale for anxiety and pain instead of a standardized assessment [13]. Currently, the *criterion standard* for evaluating pediatric anxiety during induction of anesthesia is indeed the YPAS [8,16]. Since YPAS has been implemented, further advancements with the YPAS-Short Form (YPAS-SF) are now in use, and Stewart et al. used this version [15].

Study #1 Is tablet-based interactive distraction effective on pain and anxiety during circumcision in children? A randomized controlled trial

Seventy patients aged 8 to 12 years undergoing a scheduled circumcision with local anesthesia were randomized and either given a TAB (*n *= 35) or no intervention (*n *= 35) 10 minutes before the procedure. The participants were then allowed to choose one of the three games previously downloaded on the TAB; afterward, they returned the TAB to the nurses. The TAB was returned to the patient, who was allowed to play their game five minutes before the procedure began. The outcomes of this group were compared to a control group in which the same procedure was performed without a TAB. The evaluation of both groups was completed by a blind assessor who used a Numeric Rating Scale (NRS) to assess for pain and the State-Trait Anxiety Inventory (STAI) to evaluate anxiety. The pain was evaluated on three separate occasions over the operative course, whereas anxiety was assessed two separate times. There was no significant difference found between anxiety scores before the procedure (*P* = 0.171); however, the difference in anxiety after the procedure between the two groups was significant (*P* < 0.001). The difference found among anxiety scores of the TAB distraction group before and after the procedure (*P* < 0.001) was significant. Additionally, the TAB distraction group's satisfaction scores were significantly higher than the control group's (*P* < 0.001) [13].

Study #2 Single-blinded randomized controlled study on use of interactive distraction versus oral midazolam to reduce pediatric preoperative anxiety, emergence delirium, and postanesthesia length of stay

One hundred and two patients aged 4 to 12 years undergoing outpatient surgery were randomized into a TAB (iPad) or MDZ group. Following randomization, 51 patients were given an iPad mini one minute before being separated from their parents. For the MDZ group, the 51 patients were administered 0.3 mg/kg of MDZ (20 mg maximum dose) 15-45 minutes before being separated from their parents. The TAB games these children played were pre-selected by a child life specialist. Anxiety was evaluated for both groups using a m-YPAS-Short Form (m-YPAS-SF). A preoperative nurse, PACU nurse, child life specialist, and the medical director of anesthesia each performed their assessment using the m-YPAS-SF. In addition to anxiety, the emergence of delirium, caregiver satisfaction, and the length of post-anesthesia stay were measured. Between the TBID and MDZ, the total average m-YPAS-SF scores were significantly lower in the experimental TBID group at parental separation (*P* = 0.006) and mask induction (*P* < 0.001). A significantly reduced emergence of delirium (*P* = 0.001) was found 15 minutes postemergence of anesthesia in the TBID group than in the oral MDZ group.

For the emergence of delirium, a significantly lower difference (*P* = 0.001) was found 15 minutes postemergence in the TBID group than in the oral MDZ group. As for caregiver satisfaction, the TBID group and the MDZ group produced similar results of being satisfied overall with no statistical difference. The time from PACU arrival to discharge was reduced significantly by 25 minutes (*P* < 0.001) with TBID compared to MDZ [15].

Study #3 Children and parental anxiolysis in paediatric ambulatory surgery: a randomized controlled study comparing 0.3 mg kg-1 midazolam to tablet computer based interactive distraction

A total of 118 patients aged 4 to 10 years were randomized into the TAB group (*n *= 60) or an MDZ group (*n *= 58). These patients were scheduled to undergo a variety of minor outpatient surgical procedures. Upon arrival at the surgical ward, the MDZ group received a dose of MDZ (0.3 mg/kg) 20-30 minutes before being administered anesthesia. The TAB group was given a TAB 20 minutes before anesthesia and encouraged to play games until loss of consciousness. Patient and parental anxiety were evaluated by independent psychologists using m-YPAS and STAI, respectively. Anxiety scores were taken at four different times: upon arrival to the surgery ward (time 1), separation from parents (time 2), mask induction (time 3), and postoperatively after the emergence from anesthesia back in the surgical ward (time 4). In addition to patient anxiety, nurse and parental satisfaction was graded with a verbal score from 0 (not satisfied) to 10 (highly satisfied). This study showed no significant difference in the m-YPAS score at the time of mask induction (*P* = 0.99) or parental separation. However, the mean level of the m-YPAS score spanning over all four measurements (times 1-4) was significantly reduced (*P* = 0.03) in the TAB group. TAB use also showed a significant increase in nurse satisfaction (*P* < 0.0001) and parental satisfaction (*P* = 0.04) when compared to MDZ. Marechal et al. posed that this difference could be due to a possible measurement bias because satisfaction analysis was not blinded [14].

Study #4 Tablet-based interactive distraction (TBID) vs oral midazolam to minimize perioperative anxiety in pediatric patients: a noninferiority randomized trial

One hundred and eight patients aged 1 to 11 years undergoing outpatient surgical procedures were randomly divided into a TAB group (*n* = 57) and an MDZ group (*n* = 51). The TAB group was given a TAB upon arrival to preop and was prompted to select an age-appropriate game. After the game was selected, the TAB was taken from the child and later returned one minute before parental separation and allowed to play their chosen game until induction. The children participating in the MDZ group were given their MDZ dose (0.5 mg/kg orally; up to 20 mg max) 15 to 45 minutes before departure to the operating room (OR). The primary assessment was centered on anxiety levels from baseline, which were evaluated at parental separation and anesthesia induction. The m-YPAS was used for anxiety assessment, but Seiden et al. did not address who completed the evaluation. Secondary outcomes from this study are the emergence of delirium, parental perceived anxiety, parental satisfaction, time to discharge, and post-hospitalization behavior change. The results of this study showed that TAB was significantly superior at reducing the m-YPAS score (less anxiety) at parental separation (*P* = 0.003). The authors also mentioned that this finding was further strengthened by the perceived parental anxiety results, which showed that 30 parents in the TAB group stated their children were not anxious compared to 15 parents in the MDZ group (*P* = 0.06). Compared to the MDZ group, the TAB group did not have a reduced m-YPAS score at the time of anesthetic induction (*P* = 0.04). The TAB group had significantly lower absolute emergence delirium scores compared to the MDZ group (*P* < 0.001). Time to PACU discharge was significantly higher in the MDZ group (*P* = 0.03). Post-hospitalization behavior changes between the two groups were not statistically significant. For parental satisfaction, 81% of the parents in the TAB group reported being *very satisfied* with the child separation process versus only 59% of parents in the MDZ group (*P *= 0.02) [1].

Study #5 Distraction with a hand-held video game reduces pediatric preoperative anxiety

This trial included 112 children aged 4 to 12 years undergoing general anesthesia for elective surgery. These patients were randomly assigned to one of the following three groups: parental presence (PP), parental presence plus MDZ (PPM, 0.5 mg/kg orally), or parental presence plus a handheld video game (PPVG). Before the randomization, a research nurse conducted a structured interview using the m-YPAS to establish a baseline level of anxiety (time 1; T1). After randomization, the surgical patients and their families were transferred to private cubicles in the surgery center before being transferred to the OR. At this point, parents were given written instructions regarding expectations and appropriate OR attire. Although Patel et al. did not note the exact timing of the video game or MDZ administration, the PPM and PPVG groups were given their intervention simultaneously. For the PPVG group, all the video games were self-selected from 10 games rated *E* for *everyone*. The children and their parents were escorted to the OR at least 20 minutes after being given their intervention. The children assigned to the PPVG group were allowed to play their video game through the introduction of the anesthesia mask. As the surgical team set up, an independent observer performed a second m-YPAS (time 2; T2). Once the child was anesthetized, the parents were escorted out and asked to complete a parent satisfaction survey. The results of this study showed that between the three groups, the initial (baseline; T1) m-YPAS was significantly higher in the PPM group compared to the other two groups. When comparing the baseline anxiety (T1) to anxiety at mask induction (T2), the PP group and the PPM group demonstrated a significant increase in anxiety (*P* < 0.01). Patel et al. further broke down the changes in the m-YPAS score for each of the three groups into specific age ranges. The investigators concluded their study by stating that under their set conditions, children 4-12 years who played handheld video games had less anxiety at induction of anesthesia compared to children who only had their parents present [2].

Discussion

The purpose of this systematic review is to provide a comprehensive analysis of previous studies that have investigated the use of TBID as a means of minimizing preoperative anxiety. Based on the results from this systematic review, an evidence-based consensus can be established to clarify whether TBID is effective and, if so, under what circumstances allow maximum results to be achieved. As a result, simple guidelines can be established that guide clinicians in implementing TBID, which leads to the greatest likelihood of success and ultimately improves patient care.

The following information refers to Table 2 and a number assigned to the study for reference based on the publication date. The m-YPAS is the standard for evaluating pediatric anxiety during anesthesia induction. Therefore, the m-YPAS score will be used to describe anxiety or anxiety levels.

The combined evidence from the studies in this review suggests that, in some way, TBID can reduce anxiety during a surgical course. Four of the five studies in this review demonstrated that TBID either reduced preoperative anxiety (2,4) or, at minimum, did not increase anxiety during the induction of anesthesia (1,5). Marechal et al. found that the average m-YPAS scores (anxiety levels) over four measurements were decreased in the TAB group [14]. This suggests that from arrival to the surgery ward to returning home after surgery, the children using TBID had significantly less anxiety. Similarly, Gezginci et al. [13] found that anxiety after the completed procedure was reduced with the implementation of TBID. Parental satisfaction was higher in TBID groups in each study except for Stewart et al. (2), which showed no significant difference between TBID and MDZ use [15]. Interestingly, the two study groups that measured the emergence of delirium (2,4) in their evaluations came to the same conclusion; TAB use significantly lowered the emergence of delirium postoperatively (PAED score).

**Figure 2:**
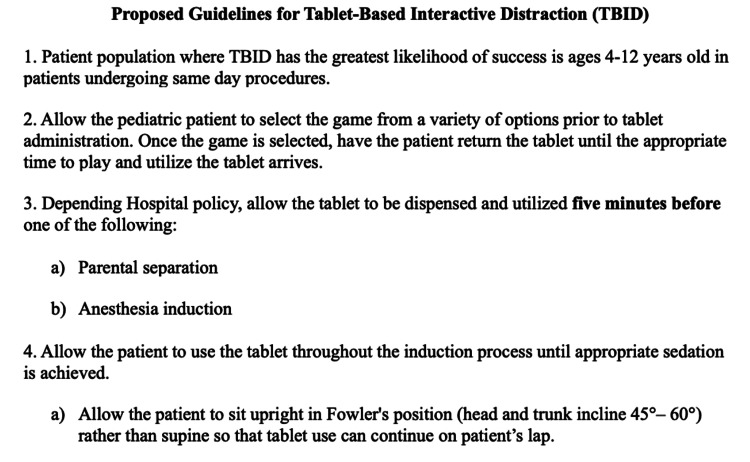
Proposed guidelines for TBID extrapolated from the systematic review.

Although specifications for implementing TBID are flexible and do not require strict instructions to achieve success, this systematic review highlights specific operational and logistical patterns that contribute to the overall success of TBID. Careful examination of each study allows one to recognize these patterns. This review permits the combination of successful patterns while leaving out other patterns that could have hindered the extent of success in previous TBID studies. For instance, the timing of TAB administration plays a vital role, with the most significant reductions in anxiety observed when the TAB is introduced to the child within five minutes before induction or before separation from parents. Allowing children to select their games from various pre-downloaded options enhances the distraction element of TBID and augments its effectiveness by keeping the patient’s attention engaged while mitigating habituation. Based on the results gleaned from the studies in this systematic review, we have proposed guidelines that can serve as a clinical plan of action for those who wish to implement TAB use. The guidelines are summarized in Figure 2 and discussed later.

The most impactful factor for successful TBID seen in the literature is the timing of TAB administration. In studies for which preoperative anxiety was significantly reduced (2,4), a TAB was given to the pediatric patient within 5 minutes before surgery or parental separation. The former of the two studies (4) administered the TAB to the children one minute before parental separation. Marechal et al. (5) distributed the TABs to the children 20 minutes before receiving anesthesia, and although the average anxiety decreased across four measurements, the preoperative anxiety was not significantly reduced [14]. Therefore, we surmise that when TABs are given five minutes before parental separation or anesthesia induction, TBID is more effective. However, it has been posited that longer times between TAB administration and parental separation or anesthesia induction could be less effective due to habituation [17].

Game selection is an essential factor contributing to the distraction effect provided by TAB use. All the studies in this review allowed the participants themselves to select a game from various pre-downloaded options. Patel et al. suggested that when children were given the liberty to pick their own game, it helps "prevent boredom and keep attentional processes engaged" [2]. Therefore, when applying TBID, it is hypothesized that allowing the children to select their own game bolsters distraction and overall success.

The articles in this systematic review provide little guidance about the intricate details of TBID. However, this review extrapolates these minor details from each study and combines them to help with the practicality of TBID (Figure 2). Seiden et al. suggested that medium-sized TABs with a durable case will improve handling, give TABs some protection, and allow the TABs to be rapidly sanitized after use [1]. Seiden et al. continued to provide logistical details about TAB use by suggesting that "during induction, it is often easier for gameplay for the child to sit upright and cross-legged as opposed to supine, allowing them to hold the TAB in their lap" [1]. Additionally, as a word of caution, patients should not use the TAB during induction in the supine position. This helpful suggestion can prevent the TAB from being dropped on the patient's face as they lose consciousness. Although these details are optional for clinicians to implement TAB use, these recommendations can help TBID be more practical and safe.

## Conclusions

Based on the complete analysis of this systematic review regarding the use of TBID to minimize preoperative anxiety in pediatric patients, the conclusion can be drawn that TBID is a practical non-pharmacological approach. Our analysis suggests that TBID modulates overall anxiety in pediatric patients aged 4 to 12 years who undergo same-day procedures. The key findings from the studies included in the review indicate that TBID may reduce preoperative anxiety, decrease the emergence of delirium, shorten the PACU stay time, and increase patient and parental satisfaction.

Despite the benefits, this review shows some variability in anxiety evaluation. YPAS is often used and is currently considered the gold standard. Interestingly, the studies mentioned in this review use various evaluators, including preoperative and PACU nurses, child life specialists, medical directors of anesthesia, blind evaluators, clinicians, independent observers, and psychologists. This variation of evaluators underscores the need for standardization in evaluating pediatric anxiety to understand TBID’s impact better. Future research should aim to assess if the perceived anxiety in patients using TBID is uniform or not among the individuals who are completing the anxiety evaluations.
